# Clinical Predictors of Immune Reconstitution following Combination Antiretroviral Therapy in Patients from the Australian HIV Observational Database

**DOI:** 10.1371/journal.pone.0020713

**Published:** 2011-06-02

**Authors:** Reena Rajasuriar, Maelenn Gouillou, Tim Spelman, Tim Read, Jennifer Hoy, Matthew Law, Paul U. Cameron, Kathy Petoumenos, Sharon R. Lewin

**Affiliations:** 1 Department of Medicine, Monash University, Melbourne, Victoria, Australia; 2 Faculty of Medicine, University Malaya, Kuala Lumpur, Federal Territory, Malaysia; 3 Centre for Population Health, Burnet Institute, Melbourne, Victoria, Australia; 4 National Centre in HIV Epidemiology and Clinical Research, Sydney, New South Wales, Australia; 5 Melbourne Sexual Health Centre, Melbourne, Victoria, Australia; 6 Infectious Disease Unit, The Alfred Hospital, Melbourne, Victoria, Australia; 7 Centre for Virology, Burnet Institute, Melbourne, Victoria, Australia; Institut National de la Santé et de la Recherche Médicale, France

## Abstract

**Background:**

A small but significant number of patients do not achieve CD4 T-cell counts >500cells/µl despite years of suppressive cART. These patients remain at risk of AIDS and non-AIDS defining illnesses. The aim of this study was to identify clinical factors associated with CD4 T-cell recovery following long-term cART.

**Methods:**

Patients with the following inclusion criteria were selected from the Australian HIV Observational Database (AHOD): cART as their first regimen initiated at CD4 T-cell count <500cells/µl, HIV RNA<500copies/ml after 6 months of cART and sustained for at least 12 months. The Cox proportional hazards model was used to identify determinants associated with time to achieve CD4 T-cell counts >500cells/µl and >200cells/µl.

**Results:**

501 patients were eligible for inclusion from AHOD (n = 2853). The median (IQR) age and baseline CD4 T-cell counts were 39 (32–47) years and 236 (130–350) cells/µl, respectively. A major strength of this study is the long follow-up duration, median (IQR) = 6.5(3–10) years. Most patients (80%) achieved CD4 T-cell counts >500cells/µl, but in 8%, this took >5 years. Among the patients who failed to reach a CD4 T-cell count >500cells/µl, 16% received cART for >10 years. In a multivariate analysis, faster time to achieve a CD4 T-cell count >500cells/µl was associated with higher baseline CD4 T-cell counts (p<0.001), younger age (p = 0.019) and treatment initiation with a protease inhibitor (PI)-based regimen (vs. non-nucleoside reverse transcriptase inhibitor, NNRTI; p = 0.043). Factors associated with achieving CD4 T-cell counts >200cells/µl included higher baseline CD4 T-cell count (p<0.001), not having a prior AIDS-defining illness (p = 0.018) and higher baseline HIV RNA (p<0.001).

**Conclusion:**

The time taken to achieve a CD4 T-cell count >500cells/µl despite long-term cART is prolonged in a subset of patients in AHOD. Starting cART early with a PI-based regimen (vs. NNRTI-based regimen) is associated with more rapid recovery of a CD4 T-cell count >500cells/µl.

## Introduction

Most patients receiving suppressive cART will experience significant increases in CD4 T-cell counts [1], [2]. In most studies, the pattern of change in CD4 T-cells following cART includes a rapid increase in CD4 T-cells in the initial three months [3], [4], [5] which is then followed by a slower increase in CD4 T-cells in the subsequent 2–3 years [4], [6], [7], [8], [9], [10], [11]. After 2–3 years of cART, changes in CD4 T-cells are less predictable. Some studies have reported sustained increases in CD4 T-cell numbers up to 4 years following suppressive cART [2], [4], [12], while others have reported a plateau beyond 3–4 years of cART [6], [7], [9], [10], [13]. In most patients, a plateau in CD4 T-cells occurs within the normal range of CD4 T-cells [2], however, in a small but significant number of patients CD4 T-cells plateau below the normal threshold of 500 cells/µl [1], [14]. There is now growing evidence that patients with CD4 T-cell counts <500 cells/µl are at an increased risk of AIDS and non-AIDS defining illnesses, despite achieving complete viral suppression on cART [15], [16], [17], [18].

Multiple cohort studies have assessed factors associated with CD4 T-cell recovery following cART and have found that older age [1], [4], [6], [8], [13], [19], [20], lower baseline CD4 T-cell counts [3], [6], [8], [12], [13], [21], higher baseline HIV RNA [3], [5], [6], [10], [14], [22], [23], [24], reduced thymic function [25], [26], increased levels of T-cell activation [27], [28] and detectable viremia while on treatment [3], [4], [6], [12], [22] are all associated with reduced CD4 T-cell recovery. Many of these studies have followed changes in CD4 T-cells in large cohorts [2], [3], [4], [6], [7], [9], [12], [13], [21], [22], [24], but few studies have had prolonged follow-up (>10 years) upon cART. The methodology used in these previous studies was unable to distinguish subgroups of patients who take a longer time to achieve CD4 T cells >500 cells/µl including those initiating cART at low baseline CD4 T-cell counts [1], from those who have plateaued at CD4 T-cell counts below 500 cells/µl and were unlikely to ever achieve this threshold. In addition, many prior cohort studies included patients who were treatment experienced prior to initiation of an effective cART regimen [1], [6], [11], [21], [24] and restricted their analysis to only include patients who have maintained viral suppression (defined differently from <50 to <1000 copies/ml) throughout follow-up [1], [8], [9], [12], [13], [14]. Though this approach measures the maximal capacity of immune recovery in patients achieving the best possible virologic outcome with cART, the findings from these studies may not be generalisable to clinical practice where treatment responses may be variable and the occurrence of virologic failure is unpredictable.

Given the clinical significance of achieving CD4 T-cell counts >500 cells/µl in HIV-infected patients and the relative limitations of some prior studies to identify patients who are unlikely to reach this threshold especially following prolonged treatment, the aim of this study was to identify factors associated with the time taken to reach CD4 T-cell counts >500 cells/µl following long-term cART in a large prospective clinic-based cohort with prolonged follow-up.

## Methods

### Patient selection

The study population consisted of all patients enrolled in the Australian HIV Observational Database (AHOD) at the time of study, n = 2853 (data updated March 2009). AHOD is an observational database that collects demographic and HIV treatment-related data from 27 sites consisting of general practitioner services, sexual health clinics and hospitals throughout Australia. The protocol for recruitment to AHOD was approved by the institutional review board of each recruiting site (listed in acknowledgements). All patients provided written informed consent prior to AHOD recruitment and no further consent was required for the conduct of this study. Details of this observational cohort have been described elsewhere [29]. For this study, patients were selected if they fulfilled the following inclusion criteria; men or women aged at least 18 years and were ART naïve at commencement of cART (defined as at least three antiretroviral drugs), treatment was initiated at CD4 T-cell counts <500 cells/µl and patients achieved controlled viral suppression (defined as HIV RNA<500 copies/ml) by 6 months of treatment initiation and maintained viral suppression for at least 12 months.

Demographic and clinical parameters such as date of birth, sex, hepatitis B and C status, HIV exposure category, diagnosis of AIDS-defining illness (ADI) before cART initiation or at follow-up, initial cART treatment regimen and all CD4 T-cell counts and HIV RNA measures from cART initiation to the most recent data available, were obtained from the March 2009 updated AHOD database. Only CD4 T-cell counts with date matched HIV RNA measures (within 1 month) were included in the analysis. The closest matched CD4 T-cell count and HIV RNA measure prior (within 1 year) to the date of cART commencement were considered as baseline values. Patients who could not be assigned a baseline CD4 T-cell count or HIV RNA measure by this definition were excluded.

### Statistical analysis

Survival analysis was used to identify determinants of CD4 T-cell recovery following cART. Two clinically relevant end-points were used to define the outcome, which were time taken to achieve CD4 T-cells counts >500 cells/µl and >200 cells/µl [30], [31]. HIV RNA for all patients was included as a time-dependent co-variate. This meant patients who were initially aviremic (based on inclusion criteria) but subsequently developed virological failure (two consecutive HIV RNA>500 copies/ml) during follow-up remained in the analysis with appropriate model adjustments for the influence of this parameter on the outcome of interest. The Cox proportional univariate model was used to initially identify candidate predictors of immune reconstitution (p<0.2). These candidate variables were then included in the Cox proportional multivariate model to identify independent predictors of recovery. A p-value of <0.05 in the multivariate model was considered significant. Finally, Schoenfeld residuals were used to assess the model for violations of the proportional hazard assumptions and the model was accepted only after no violations were shown to occur. Baseline CD4 T-cell counts were square transformed in order to meet the proportional hazard assumption in the analysis of time to achieve CD4 T-cell counts >500 cells/µl. Comparisons of survival curves of patients achieving CD4 T-cell counts >500 cells/µl were done using the Log-rank test. All statistical analyses were performed using STATA (version 10).

## Results

### Cohort characteristics

**Figure 1:**
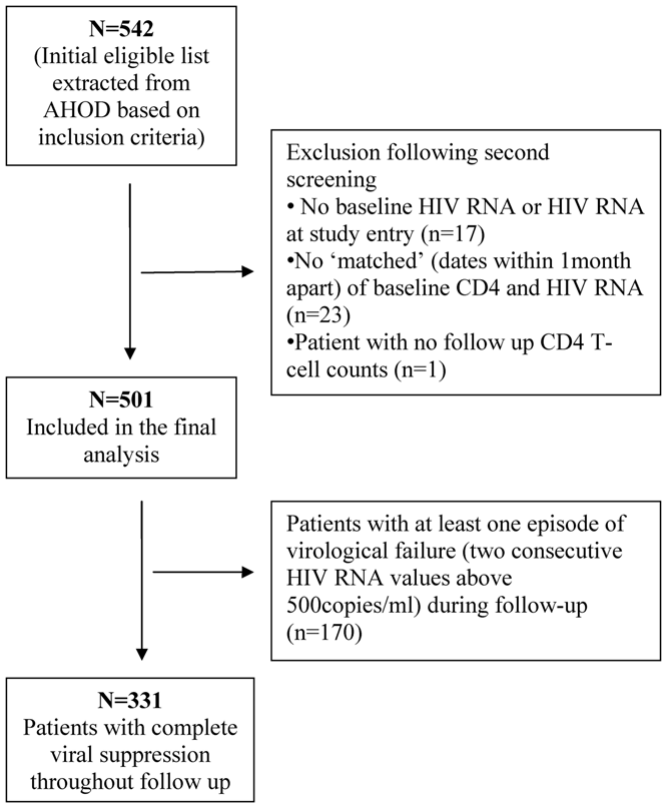
Patient disposition.

Five hundred and forty two (19%) patients fulfilled the inclusion criteria and were selected from a study population of 2853. The remainder of AHOD (n = 2311) were excluded for the following reasons; treatment experienced (n = 1599; receiving mono or dual therapy prior to cART), initiated treatment at CD4 T-cell counts >500 cells/µl (n = 424) and did not achieve controlled viremia by 6 months or sustain viral suppression for at least 12 months post-cART (n = 288). Of the 542 patients who fulfilled the inclusion criteria, 41 patients were excluded because they had no matched baseline CD4 T-cell/HIV RNA measure recorded in the database or had no recorded baseline HIV RNA or follow up CD4 T-cell counts (Figure 1). The remaining 501 patients were included in the final analysis.

The majority of patients were males (94%) and the median (IQR) age at cART initiation was 39 (32–47) years (Table 1). The median (IQR) baseline CD4 T-cell count was 236 (130–350) cells/µl and HIV RNA levels were 88 050 (25 875–250 070) copies/ml. Sixty seven patients (13%) reported a history of ADI prior to the commencement of cART while 23 patients (4.6%) developed 29 episodes of ADI on cART. These episodes mostly occurred soon after cART initiation (median (IQR) = 4.5 (0.9–38.4) months). The median (IQR) follow-up duration for the cohort was 6.5 (3.4–10.2) years and a median of 22 (IQR 12–34) pairs of matched CD4 T-cell and HIV RNA measures per patient were included in the analysis. Nelfinavir and indinavir were the most commonly used PIs (32% and 27% of all PI regimens respectively). Most recruitment to AHOD was before 2005, when these less potent PIs were being widely used in Australia. Nevirapine and efavirenz were used equally (45% and 55% of all NNRTI regimens).Thirty-four percent of patients developed at least one episode of virological failure (VF) during the follow-up period (5 and 10-year cumulative incidence (95% CI) to the first episode of VF was 8.1/100 person-years (100 pyr) (6.9–9.6) and 7.0/100 pyr (6.0–8.1) respectively). Among the patients who developed episodes of VF, the median (IQR) proportion of time spent with HIV RNA>500 copies/ml in relation to the total duration of observation for each patient was 20 (7–38) %. Seventeen (3.4%) patients died during the follow-up period (5 and 10-year cumulative incidence (95% CI) to death was 0.2/100 pyr (0.1–0.6) and 0.5/100 pyr (0.3–0.8) respectively).

**Table 1:** Demographic and clinical characteristics of patients from AHOD who met the inclusion criteria for this study.

Patient characteristics	All patients (n = 501) Median (IQR)	Patients with complete viral suppression (n = 331) Median (IQR)
**Gender**		
**-** Male, n (%)	470 (93.8%)	310 (93.7%)
**-** Female, n (%)	30 (6.0%)	21 (6.3%)
**-** Transgender, n (%)	1 (0.2%)	0
**Age at cART initiation (years)**	39 (32.0–47.0)	41 (34.0–48.0)
**Positive hepatitis C virus antibody, n (%)**	41 (8.2%)	23 (6.9%)
**Positive hepatitis B surface antigen, n (%)**	26 (5.2%)	14 (4.2%)
**Baseline CD4+ T-cell count (cells/µl)**	238 (130–350)	234 (130–340)
**Baseline HIV RNA (copies/ml)**	87 800 (25 650–248 450)	85 600 (23 000–220 000)
**HIV exposure category**		
**-** MSM	376 (75.1%)	247 (74.8%)
**-** Heterosexual contact	66 (13.1%)	44 (13.3%)
**-** Others*	59 (11.8%)	40 (13.0%)
**Follow-up duration, years**	6.5 (3.4–10.2)	5.0 (2.7–9.3)
**History of AIDS defining illness, n (%)**	67 (13.3%)	44 (13.3%)
**Death during follow-up, n (%)**		
**-** AIDS/HIV-related	4 (0.8%)	2 (0.6%)
**-** Not HIV-related	9 (1.8%)	6 (1.8%)
**-** Unknown	4 (0.8%)	1 (0.3%)
**cART regimen at baseline, n (%)**		
-NNRTI-based	293 (58.4%)	202 (60.8%)
-PI-based	179 (35.7%)	106 (31.9%)
-Triple NRTI-based	20 (4.2%)	15 (4.8%)
-Others	9 (1.8%)	8 (2.4%)

All parameters are median (IQR) unless otherwise stated.

cART-combination Antiretroviral therapy; MSM – men who have sex with men; NNRTI – non-nucleoside reverse transcriptase inhibitor; PI – protease inhibitor (boosted and unboosted); NRTI – nucleoside reverse transcriptase inhibitor.

*Others included injecting drug use, transfusion and unrecorded.

PIs included Atazanavir (boosted and unboosted) (5%), Lopinavir/Ritonavir (11%), Indinavir (boosted and unboosted) (32%), Nelfinavir (27%), Ritonavir (10%), Saquinavir (boosted and unboosted) (13%), Tipranavir (1%), Fosamprenavir (1%); NNRTIs included Delavirdine (1%), Efavirenz (44%), Nevirapine(55%);

### Time taken to achieve CD4 T-cell counts >500 cells/µl with long-term cART

**Figure 2:**
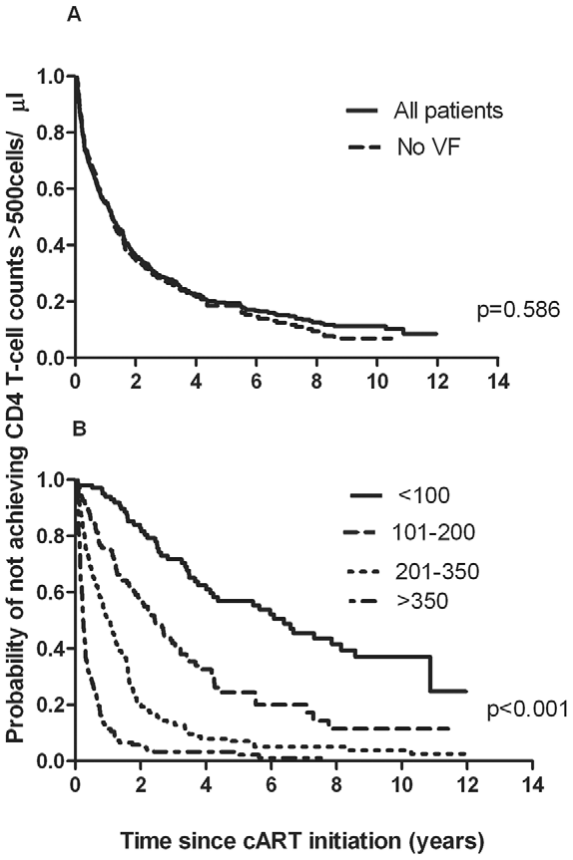
Kaplan-Meier plots showing time taken to achieve CD4 T-cell counts >500 cells/µl following cART initiation. Kaplan-Meier plots showing time taken to achieve CD4 counts >500cells/µl among (A) patients in the total cohort (n = 501) and patients with no evidence of virological failure (VF) (2 consecutive HIV RNA>500copies/ml) throughout follow-up (n = 331) and (B) all patients (n = 501) stratified by baseline CD4 T-cell counts (<100cells/µl, n = 99; 101–200cells/µl, n = 107; 201–350cells/µl, n = 172; >350cells/µl, n = 123). Comparisons of survival curves were done using the Log-rank test (STATA 10.0).

The majority of patients (80%) eventually reached CD4 T-cell counts >500 cells/µl and the median time taken to achieve this was variable 1.2 (IQR = 0.3–2.6) years. The majority of patients (75%) who reached CD4 T-cell counts >500 cells/µl in the cohort, achieved this threshold within 3 years of initiating cART (Figure 2A) but a subset of patients took longer. These findings did not change when the analysis was restricted to patients who showed no evidence of virological failure (defined as 2 consecutive HIV RNA>500 copies/ml) during follow-up, indicating that slower reconstitution in these patients was unlikely to be due to loss of virological control. As previously shown, stratification of the time taken to achieve CD4 T-cell counts >500 cells/µl by baseline CD4 T-cell counts (Figure 2B) showed that patients starting cART at lower baseline CD4 T-cell categories took the longest times to achieve counts >500 cells/µl [1].

### A small subset of patients fail to achieve CD4 T-cell counts >500 cells/µl despite long-term cART

Twenty percent of patients failed to achieve CD4 T-cell counts >500 cells/µl. A similar proportion was found in the subset of patients who achieved complete viral suppression throughout follow-up (n = 331, Figure 2A, characteristics described in Table 1). Of the patients who failed to achieve CD4 T-cells >500 cells/µl despite good virological control (n = 67), 25% received at least 5 years of continuous suppressive therapy while 8% had been on therapy for more than 10 years.

The 5-year cumulative incidence (95% CI) of not achieving CD4 T-cell counts >500 cells/µl in patients initiating cART with baseline CD4 T-cell counts of <100, 101–200, 201–350 and >350 cells/µl was 7.3/100 pyr (4.9–10.9), 10.2/100 pyr (6.9–15.0), 5.8/100 pyr (3.4–10.1) and 1.5/100 pyr (0.2–10.7) respectively (Figure 2B). The 5-year cumulative incidence (95% CI) of patients not achieving counts >500 cells/µl was also higher among those initiating cART with a NNRTI-based regimen (9.1/100 pyr (6.8–12.1)) compared to patients initiating therapy with PI-based regimens (boosted and unboosted; 4.0/100 pyr (2.3–7.0).

### Predictors of time taken to achieve CD4 T-cell counts >500 cells/µl and >200 cells/µl following long-term cART

In a univariate analysis, factors associated with a more rapid time to achieve CD4 T-cell counts >500 cells/µl included higher baseline CD4 T-cells (square-transformed) (HR 1.14, 95% CI 1.13–1.16, p<0.001), younger age at cART initiation (HR 0.99 95% CI 0.98–0.99, p = 0.023), no history of ADI prior to cART initiation (HR 0.45, 95% CI 0.33–0.63, p<0.001) and earlier calendar year of cART initiation (HR 0.77, 95% CI 0.61–0.97, p = 0.024) (Table 2). Although HIV exposure category, HIV RNA measure as a time dependent covariate and treatment regimen were not significant in the univariate model, these parameters were still included in the multivariate model because they were considered candidate predictors (p<0.2) based on our pre-defined analysis strategy.

**Table 2:** Predictors of time taken to achieve CD4 T-cells >500 cells/µl (n = 501).

Variable	Univariate Hazard Ratio (95% CI)	p-value	Multivariate Hazard Ratio (95% CI)	p-value
**CD4 T-cell baseline (square-transformed), per 100 units** #	1.14 (1.13–1.16)	<0.001	1.15 (1.13–1.16)	<0.001
**Baseline HIV RNA, per 100 000 copies/ml**	0.99 (0.97–1.02)	0.606		
**HIV RNA per 100 000 copies/ml (time dependent covariate)**	0.33 (0.09–1.12)	0.074		
**Age at cART initiation, years**	0.99 (0.98–0.99)	0.023	0.99 (0.98–0.998)	0.019
**Initial cART regimen**				
-NNRTI regimen*	-		-	
-PI regimen	1.16 (0.94–1.42)	0.158	1.24 (1.01–1.52)	0.043
-Triple NRTI regimen	1.10 (0.66–1.83)	0.716	1.08 (0.65–1.81)	0.763
-Others	1.40 (0.69–2.84)	0.349	0.77 (0.38–1.57)	0.476
**Calendar year of starting cART**				
-1997–1999*	-			
-2000–2004	0.77 (0.61–0.97)	0.024		
-≥2005	0.93 (0.70–1.23)	0.607		
**HIV exposure category**				
-MSM*	-			
-Heterosexual contact	0.74 (0.54–1.00)	0.051		
-Other**	0.92 (0.67–1.27)	0.610		
**HBsAg positive**				
-Yes	0.88 (0.56–1.38)	0.563		
-No*	-			
**HCV Ab positive**				
-Yes	0.81 (0.56–1.17)	0.256		
-No*	-			
**Gender**				
-Male	0.90 (0.61–1.33)	0.604		
-Female*	-			
**History of ADI**				
-Yes	0.45 (0.33–0.63)	<0.001		
-No*	-			

*Reference category.

**Others included injecting drug use, transfusion and unrecorded.

# For every 100-unit increase in square transformed baseline CD4 T-cell counts, the hazard of achieving a CD4 T-cell threshold of >500cells/µl increased by 15%.

cART-combination Antiretroviral therapy; MSM – men who have sex with men; NNRTI – non-nucleoside reverse transcriptase inhibitor; PI – protease inhibitor (boosted and unboosted) ; NRTI – nucleoside reverse transcriptase inhibitor; ADI – AIDS-defining illness; HBsAg – hepatitis B surface antigen; HCV Ab – hepatitis C antibody.

In the multivariate model, only higher baseline CD4 T-cell counts (square-transformed) (HR 1.15, 95% CI 1.13–1.16, p<0.001), younger age (HR 0.99, 95% CI 0.98–0.998, p = 0.019) and initiating treatment with a PI-based regimen (boosted and unboosted) (vs NNRTI-based regimen) (HR 1.24, 95% CI 1.01–1.52, p = 0.043) were significantly associated with time taken to achieve CD4 T-cells >500 cells/µl. A prior history of ADI and calendar year of starting cART were no longer significant in the multivariate model.

In a subset of patients initiating cART at CD4 T-cell counts <200 cells/µl, we assessed time taken to achieve CD4 T-cells >200 cells/µl (n = 196). The median (IQR) baseline CD4 T-cells for these patients was 110 (50–170) cells/µl. In this multivariate analysis, more rapid recovery to CD4 T-cell counts >200 cells/µl was significantly associated with a history of ADI (HR 1.53, 95% CI 1.07–2.18, p = 0.018), higher baseline CD4 T-cell count (HR 4.53, 95% CI 3.32–6.19, p<0.001) and higher baseline HIV RNA (HR 1.07, 95% CI 1.05–1.10, p<0.001) (Table 3).

**Table 3:** Predictors of time taken to achieve CD4 T-cells >200 cells/µl (n = 196).

Variable	Univariate Hazard Ratio (95% CI)	p-value	Multivariate Hazard Ratio (95% CI)	p-value
**CD4 T-cell baseline, per 100 cells/µl**	3.35 (2.55–4.40)	<0.001	4.53 (3.32–6.19)	<0.001
**Baseline HIV RNA, per 100 000 copies/ml**	1.04 (1.01–1.07)	0.012	1.07 (1.05–1.10)	<0.001
**HIV RNA, per 100 000 copies/ml (time dependent covariate)**	0.80 (0.46–1.39)	0.425		
**Age at cART initiation, years**	0.99 (0.98–1.01)	0.271		
**Initial cART regimen**				
-NNRTI regimen*	-			
-PI regimen	1.07 (0.79–1.44)	0.666		
-Triple NRTI regimen	0.51 (0.23–1.12)	0.093		
**Calendar year of starting cART**				
-1997–1999*	-			
-2000–2004	0.96 (0.70–1.32)	0.802		
-≥2005	1.24 (0.84–1.83)	0.275		
**HIV exposure category**				
-MSM*	-			
-Heterosexual contact	0.71 (0.47–1.06)	0.091		
-Others**	0.64 (0.41–1.01)	0.055		
**HBsAg positive**				
-Yes	1.11 (0.61–2.00)	0.738		
-No*	-			
**HCV Ab positive**				
-Yes	1.04 (0.64–1.68)	0.868		
-No*	-			
**Gender**				
-Male	0.95 (0.53–1.71)	0.865		
-Female*	-			
**History of ADI**				
-Yes	0.79 (0.58–1.08)	0.138	1.53 (1.08–2.18)	0.018
-No*	-			

*Reference category.

**Others included injecting drug use, transfusion and unrecorded.

cART-combination Antiretroviral therapy; MSM – men who have sex with men; NNRTI – non-nucleoside reverse transcriptase inhibitor; PI – protease inhibitor (boosted and unboosted) ; NRTI – nucleoside reverse transcriptase inhibitor; ADI – AIDS-defining illness; HBsAg – hepatitis B surface antigen; HCV Ab – hepatitis C antibody.

## Discussion

CD4 T-cell recovery following cART is variable and patients who fail to achieve CD4 T-cell counts >500 cells/µl remain at risk of AIDS and non-AIDS defining illnesses. In a large prospective clinic-based cohort, we found that 80% of patients who achieved early (12 months post-ART) virological control following cART, achieved a CD4 T-cell count >500 cells/µl within a median follow-up of 1.2 years. However, there was a subset of patients who took significantly longer to eventually reach a CD4 T-cell count of 500 cells/µl, independent of whether there was complete viral suppression throughout follow-up. Twenty percent of this clinic-based cohort did not achieve CD4 T-cell counts >500 cells/µl after a median follow-up of 6.5 years. In a multivariate analysis, we found faster immune reconstitution to CD4 T-cell counts >500 cells/µl was significantly associated with higher baseline CD4 T-cell counts, younger age and initiation of cART with a PI-based (vs NNRTI-based) regimen.

Previous studies have used multiple approaches to quantify CD4 T-cell recovery following cART. Unlike most studies which have assessed CD4 T-cell recovery as the rate of CD4 T-cell increase or an absolute change in CD4 T-cell count, we quantified CD4 T-cell recovery by the time taken to achieve a CD4 T-cell count >500 cells and >200 cells/µl. We believe this approach was more robust because it accounted for both the differences in rate, pattern and extent of immune reconstitution that patients receiving cART may experience especially following long-term therapy. Additionally, this approach eliminated the need to establish a consistent pattern of CD4 T-cell increase with time, which is required when more complex regression models are used to assess CD4 T-cell recovery [9]. Regression models however, do have the advantage of estimating subject specific and average CD4 T-cell gains in the cohort and unlike the Kaplan Meier approach is less affected by the biases that may be inferred due to the loss of patients during follow-up.

We found that the time taken to achieve CD4 T-cell counts >500cells/µl was highly variable, with a small but significant subset of patients experiencing prolonged periods (>5 years) with a CD4 T-cell count <500 cells/µl. As expected this was most commonly observed in patients who initiated cART at CD4 T-cell counts <100 cells/µl. Many other studies have demonstrated poor CD4 T-cell recovery in patients initiating cART at low CD4 T-cell counts [1], [3], [13], [21] or those experiencing poor virological control [6], [22], [32], [33] following cART. We surprisingly found that in our cohort, loss of virological control during treatment did not significantly alter the distribution of time taken to achieve counts >500cells/µl, nor did it influence the proportion of patients achieving this threshold. This observation may have been influenced by the fact that our study excluded patients with poor virological response in the first 12 months following cART, a time period when CD4 T-cell increases are greatest and the occurrence of virological failure at this early phase may have the strongest negative impact on subsequent rates of CD4 T-cell increase [32]. Additionally, the median proportion of time spent with HIV RNA >500 copies/ml over the entire observation period among patients experiencing virological failure was short in this study. Other studies have described higher thresholds of detectable viremia before a significant negative influence on CD4 T-cell recovery was found to occur [6].

Even among patients who experienced continued viral suppression throughout follow-up, there was a subset of patients who took significantly longer to achieve CD4 T-cells >500 cells/µl. Similar to that described in other studies [1], [13], [14], we also found a small subset of patients failed to achieve counts >500 cells/µl despite receiving long-term suppressive cART.

We found baseline CD4 T-cell count was independently associated with the time taken to reach both CD4 counts >500 cells/µl and >200 cells/µl in both multivariate analyses. Numerous studies with long-term follow-up have also reported similar findings [3], [4], [6], [8], [13], [21]. Patients starting therapy at low CD4 T-cell nadir may experience immunologic dysfunction that may impede their capacity to achieve robust CD4 T-cell increases with cART. Longitudinal studies have shown that patients starting therapy at low CD4 T-cell nadir experience a skewed distribution of CD4 T cell subsets with significantly lower naïve and higher effector cell subsets compared to patients starting treatment at higher CD4 T-cell nadir (counts >350cells/µl) [34], [35]. This abnormality persists following CD4 T-cell recovery even after prolonged (median 6 years) suppressive therapy [35]. The greater turnover of effector cells compared to the longer-lived naïve cells may in part contribute to the slower net increase in CD4 T-cell numbers experienced by these patients following treatment. Additionally numerous studies have also found that poor CD4 T-cell recovery following suppressive cART is associated with increased immune activation (measured by soluble and T-cell activation markers) and T-cell apoptosis [36], [37], [38], [39], [40]. Taken together, these data imply that in patients starting cART at low baseline CD4 T-cell counts, numerous pathogenic mechanisms may collectively work to slow their increase in CD4 T-cell numbers and prolong their risk of acquiring AIDS and non-AIDS defining illnesses.

Current HIV treatment guidelines are mixed on when to initiate cART with some guidelines recommending initiation at <350 cells/µl [41], [42] and others recommending initiation at <500 cells/µl [43], [44]. Our data demonstrated that a significantly higher proportion of patients initiating treatment at counts >350 cells/µl achieved counts >500 cells/µl and that these patients spent shorter periods below this threshold following cART compared to patients starting therapy at CD4 T-cell counts between 200–350 cells/µl. These data provide further support for earlier initiation of cART.

As previously reported [1], [4], [6], [8], [13], [19], [20], we found younger age to be a significant predictor of faster reconstitution to counts >500 cells/µl. The favourable immune response associated with starting cART at a younger age is probably related to the T-cell regenerative capacity of these patients compared to older patients in whom physiological involution of the thymus may limit their ability to produce naïve T-cells [45], [46], [47], [48]. Younger age has also been associated with better CD4 T-cell recovery in other lymphopenic conditions including following hematopoetic stem cell transplantation and chemotherapy [49], [50], [51]. In this study, age was only significantly associated with time to reach a CD4 T-cell count >500 cells/µl and not >200 cells/µl. This was consistent with the findings from other studies [3], [12], [23], [45] where age positively influenced long-term immune reconstitution and not early recovery (<3 months) where increases in CD4 T-cell numbers immediately following cART initiation was generally associated with the redistribution of T-cells from lymphoid tissue [34], [52] rather than de novo T-cell production.

We surprisingly found that patients initiating cART with a PI-based regimen (boosted and unboosted) achieved faster reconstitution to CD4 T-cell counts >500 cells/µl when compared to patients receiving NNRTI-based regimens. This association remained significant even when calendar year was included in the multivariate model to adjust for the historical preference of initiating patients with PI-based regimens during the early cART era (data not shown). We also explored if treatment switches from the anchor regimen during follow-up had an influence on the time to reach CD4 T-cell counts >500 cells/ul by including treatment regimen as a time dependent co-variate in the model but did not find a significant association (data not shown), consistent with another recent study [53]. The lack of association between subsequent treatment switch and CD4 T-cell recovery may be because the majority of patients (86.5%) who achieved counts >500 cells/µl in this cohort did so while receiving their initial treatment regimen. The influence of treatment regimen on the capacity of long-term CD4 T-cell reconstitution is still unclear. Multiple studies have reported that patients receiving PI-based regimens have better CD4 T-cell recovery [32], [54], [55], [56], [57], [58], [59], [60] while others have not confirmed these observations [61], [62], [63], [64], [65], [66]. Protease inhibitors have been described to exert anti-apoptotic [67] and restore T-cell proliferative responses [68] independent of their antiretroviral activity, however these findings have also been inconsistent [69]. The positive association of PI-based regimen and more rapid CD4 T-cell recovery in our study should be interpreted with caution because this is an observational study of a clinic-based cohort where the choice of treatment offered to patients may not have been random and may have been influenced by multiple factors including degree of immunosuppression at the time of initiation of cART and viral mutation patterns that has not been adjusted for in our statistical analysis.

Apart from baseline CD4 T-cell counts, the factors associated with time to reach CD4 T-cell counts >200 cells/µl were found to be different from those associated with recovery to >500 cells/µl. We found that patients with a history of an ADI and higher baseline HIV RNA were associated with faster recovery to counts >200 cells/µl consistent with other reports [3], [5], [6], [12], [14], [22], [23], [24]. HIV-infected patients with profound immune-suppression may experience greater sequestration of CD4 T-cells into lymphoid tissues which are then released into the peripheral circulation following cART-induced viral suppression [45], [52], [70], [71]. This might potentially explain the faster time taken to achieve CD4 T-cell counts >200 cells/µl among patients with higher baseline HIV RNA and ADI in this study.

We did not find an association between hepatitis C serostatus and CD4 T-cell recovery as has been previously described in some [22], [61], [72], [73] but not all [74], [75], [76] studies. This could be due to the small number of patients (<10%) with HCV in this study. Additionally, HCV seropositivity is strongly associated with active injecting drug use [72] which in this study only accounted for a minority in the HIV-risk transmission group.

The major strength of our study is the long duration of patient follow-up and this characteristic of the cohort allowed us to distinguish patients with a slow recovery to CD4 T-cell counts >500 cells/µl from those who are unlikely to ever achieve this threshold. However, there were several important limitations too. First, we defined viral suppression as an HIV RNA<500 copies/ml instead of the more sensitive cut-off of <50 copies/ml [2], [77]. We did this because not all patients had testing with the more sensitive HIV RNA assays in the early cART era. Second, we used the time to reach a defined event of CD4 T-cells >500 cells/µl or >200 cells/µl which could potentially overestimate the number of patients achieving these events given the wide fluctuations in CD4 T-cell counts that may occur in a patient and it is possible that these patients may not consistently maintain CD4 T-cell counts above these thresholds over the long term. As previously reported [1], [53], we found that the likelihood of a patient achieving a threshold of >500 cells/µl or >200 cells/µl and then experiencing a decline in CD4 T-cell count was low. The majority of patients who reached these thresholds maintained these levels of CD4 T-cell reconstitution unless they experienced episodes of virological failure. In patients without episodes of virological failure, only 4 patients (1.5%) who reached counts >500 cells/µl experienced a subsequent decline in CD4 T-cell numbers without any evidence of increase in HIV RNA while all patients who reached CD4 T-cell counts >200 cells/µl subsequently maintained counts above this threshold. Finally, this was an observational clinic-based cohort and therefore our results may have been biased towards patients who reliably attend physician appointments.

In summary, we found that the majority of patients in a clinic-based observational cohort eventually achieved CD4 T-cell counts >500 cells/µl but in 8% of patients this took over 5 years. Our findings support the initiation of cART early as both younger age and higher baseline CD4 T-cell counts were associated with faster CD4 T-cell recovery to counts >500 cells/µl. The small number of patients who failed to achieve counts >500cells/µl despite receiving long-term suppressive cART suggest that current standard cART regimens alone may not be sufficient to achieve complete CD4 T-cell recovery in some patients. In these patients, other alternative immune-based therapies may need to be explored.
